# The Neuropharmacology of Implicit Learning

**DOI:** 10.2174/157015910793358178

**Published:** 2010-12

**Authors:** Julia Uddén, Vasiliki Folia, Karl Magnus Petersson

**Affiliations:** a Max Planck Institute for Psycholinguistics, Nijmegen, the Netherlands; b Stockholm Brain Institute, Karolinska Institutet, Stockholm, Sweden; c Donders Institute for Brain, Cognition and Behaviour, Centre for Cognitive Neuroimaging, Radboud University Nijmegen, Netherlands; d Institute of Biotechnology & Bioengineering/CBME, Universidade do Algarve, Faro, Portugal

**Keywords:** Implicit learning, procedural learning, neurotransmittors, neuromodulators, dopamine, serotonin, acetylcholine, noradrenalin, GABA, glutamate, NMDA, ampakines.

## Abstract

Two decades of pharmacologic research on the human capacity to implicitly acquire knowledge as well as cognitive skills and procedures have yielded surprisingly few conclusive insights. We review the empirical literature of the neuropharmacology of implicit learning. We evaluate the findings in the context of relevant computational models related to neurotransmittors such as dopamine, serotonin, acetylcholine and noradrenalin. These include models for reinforcement learning, sequence production, and categorization. We conclude, based on the reviewed literature, that one can predict improved implicit acquisition by moderately elevated dopamine levels and impaired implicit acquisition by moderately decreased dopamine levels. These effects are most prominent in the dorsal striatum. This is supported by a range of behavioral tasks in the empirical literature. Similar predictions can be made for serotonin, although there is yet a lack of support in the literature for serotonin involvement in classical implicit learning tasks. There is currently a lack of evidence for a role of the noradrenergic and cholinergic systems in implicit and related forms of learning. GABA modulators, including benzodiazepines, seem to affect implicit learning in a complex manner and further research is needed. Finally, we identify allosteric AMPA receptors modulators as a potentially interesting target for future investigation of the neuropharmacology of procedural and implicit learning.

## 1. INTRODUCTION

Everyday life provides many examples of complex behavior. One of the most intriguing complex behaviors is perhaps language communication *via* the exchange of (structured) sentences. This skill is learnt during early childhood, largely as a consequence of self-organized, non-supervised acquisition mechanisms, including implicit sequence learning and incidental learning [1]. The acquisition of complex knowledge structures, essentially without deliberate explicit strategies and supervised teaching, has been investigated under the umbrella term of implicit learning. Skill learning and procedural learning are related forms of learning and repetition priming (also called implicit memory) is likely to be related as well. The enterprise of finding the neurobiological mechanisms underlying implicit learning is still in its infancy. Some progress has been made on localization of important brain circuitry supporting this type of learning [2], but which neurotransmitters are utilized in these circuits, as well as which functional role these play is largely unknown.

In general terms, implicit learning is “the process whereby a complex, rule-governed knowledge base is acquired, largely without any requirements of awareness of either the process or the product of acquisition” [3]. More precisely, implicit learning has four characteristics: (1) no or limited explicit access to the knowledge acquired and how it is put to use; (2) the acquired knowledge is more complex than simple associations (such as simple stimulus-response associations) or exemplar specific frequency-counts; (3) it is an incidental consequence of information processing and not explicit hypothesis testing and (4) it does not rely on declarative memory mechanisms [2]. Implicit learning is typically investigated using stimuli such as patterns or structured sequences.

There is good evidence that the frontal cortex and the basal ganglia (fronto-striatal circuits) are involved in implicit learning in humans. This has been characterized in patient (lesion) studies [for reviews see e.g. 2, 4] and functional neuroimaging studies in healthy volunteers [5-7]. Likewise, there is empirical research showing that the prefrontal cortex is involved [4, 5, 8, 9]. The fact that implicit learning does not rely on declarative memory mechanisms (i.e., the medial temporal lobe, MTL, memory system) is evidenced by preserved implicit learning in amnesic patients [4, 10, 11]. There is little evidence that the MTL is engaged by implicit learning tasks [2, 4]. In contrast, in a recent study [12] the MTL was deactivated during an artificial grammar learning paradigm, an implicit learning task that we will describe below. This is consistent with the view that implicit processing does not rely on declarative memory mechanisms that engage the MTL memory system. However, there is yet no detailed account of the characteristics of the learning mechanisms involved in implicit learning, the nature of the knowledge acquired, or how this knowledge is represented and put to use, nor a detailed characterization of the neural processing infrastructure supporting these functions, for instance in terms of neurotransmittor and receptor systems [13].

In this paper we review the neuropharmacology literature relevant to implicit learning in an attempt to characterize what is known about the neuropharmacology of implicit learning. We focus mainly on neuromodulators, which are neurotransmitters, typically with slow temporal characteristics that induce temporally extended and non-local effects on synaptic transmission. In particular, we review the role of the four main neuromodulator systems in implicit learning: dopamine, serotonin, noradrenalin, and acetylcholine. Related studies on procedural and probabilistic learning are also considered. Since the functions of γ-aminobutyric acid (GABA) and allosteric α-amino-3-hydroxy-5-methyl-4-isoxazolepropionic acid (AMPA) receptor modulators (ampakines) in implicit learning are likely to be relevant, we also review these systems.

The most well-studied implicit learning task is perhaps artificial grammar learning [AGL, for reviews see 14, 15]. The task consists of a learning or acquisition phase and a test phase. In the acquisition phase, participants are exposed to an acquisition set of symbol sequences generated from a formal grammar (i.e., a complex rule system), typically in a short term memory task. In the standard version, subjects are subsequently informed that the sequences were generated according to a complex set of rules and asked to classify novel sequences as grammatical or not, typically with the instruction to base their classification decisions on their immediate intuitive judgment (i.e., guessing based on “gut feeling”). It is a robust and well-replicated finding that subjects perform significantly above chance on this type of task [5, 9, 13, 15], and more so after several days of implicit acquisition [16, 17].

Besides AGL, one of the most intensely investigated implicit learning paradigms is the serial reaction time task (SRTT) [15, 18]. In the classical version of the SRTT, implicit learning is inferred from faster reaction times when subjects respond to a fixed reoccurring sequence versus random sequences. In this task, sequences are typically displayed as digit sequences, for example of the digits 1-4. Each digit corresponds to a button press so that 1 for example means “press the button under your index finger”, 2 means “press the button under your middle finger” and so on. In our example, the implicit knowledge would consist of the reoccurring sequence pattern 3-2-4-1 that makes response times faster compared to random sequences. Again, the participants typically report no or little awareness of their acquired knowledge. This paradigm has also been implemented for investigation in rodents but the validity of implicit learning in rodent research is still controversial [19]. There are several proposals for how knowledge of sequence structure is acquired in animals, including the acquisition of stimulus–response associations or more abstract representations [19]. Generally, the learning of sequences with a fixed order can be viewed as a special case of acquiring knowledge about more general structural regularities or temporal contingencies in stimuli [2, 14].

In Tulving’s organization of human memory and learning systems, he argues that procedural (or non-declarative) learning, lumping together motor and cognitive skills as well as simple conditioning and associative memories, is acquired by implicit processes [20]. We will review some tasks which are commonly conceptualized as procedural learning tasks, including problem solving puzzles like the Tower of Hanoi, London or Toronto [21], mirror reading/drawing, and tracking tasks. These tasks do require acquisition of complex sequenced behavior. Sequential learning is a crucial component of implicit learning tasks like AGL and SRTT, but it is not necessarily a component of all procedural learning tasks. We would like to stress that depending on the exact administration of the task, procedural learning tasks may differ from implicit learning tasks in important ways. For instance, procedural learning tasks may be affected by explicit problem solving strategies, especially during the early stage of acquisition. Another potential difference is found in mirror reading and trail tracking tasks, where it is not clear whether subjects learn novel information about structured stimuli [see discussion in 4, 15].

In the classical analysis of procedural learning (or skill learning), skill acquisition is viewed as gradually becoming less controlled and more automatized, corresponding to a transition from controlled explicit to automatic implicit processing [22]. It has been suggested that parallel explicit and implicit processes might be employed for this purpose [4, 13, 23]. Further differences between procedural and implicit learning tasks are that acquisition and test phases are typically not separated in procedural learning tasks. However, the behavioral pattern and neural implementation revealed in neuropsychological studies are similar for procedural and implicit learning tasks [21, 24, 25], perhaps because of a shared implicit component. It is important to establish the robustness of neuropharmacological effects on the implicit learning system(s) through converging evidence from a range of tasks. Thus, we will include a discussion of two neuropharmacological models of automatization. We will also review studies that investigate procedural learning when the implicit component of the task seems relevant, although it is difficult to determine exactly how much explicit components might contribute to behavioral and pharmacological effects.

In this review, we have occasionally chosen to include studies using probabilistic learning tasks. We only did so if the described task fulfills all criteria for implicit learning stated above [see also the discussion in 4, 14, 15] with the exception that we allow the information acquired to be somewhat less complex than in most implicit learning tasks. The simplest form of probabilistic learning is perhaps the 2-choice probabilistic learning task. The task is to predict which of two events, A or B, will happen after a signal. Events A and B follow the signal in a random sequence, but with certain probabilities. For example, event A will occur 75% of the times and B 25% of the times. The task can also include longer sequences that occur with certain probabilities and the probabilities might be changed within a session. When the probabilities are inverted or otherwise changed, so that B becomes the more common event, this is called reversal learning. Probabilistic sequence learning has been interpreted as an implicit learning task [26]. Since the knowledge acquired in the 2-choice probabilistic learning task and variants such as gambling tasks (where there are more choices), is simpler than in probabilistic sequence learning, contamination by explicit strategies is a possibility. However, the implicit component in these tasks is probably still relevant enough for the purpose of this review.

Implicit memory is a concept that has been used in two ways in the literature, most commonly as a term exchangeable with repetition priming. It is also used as a wider concept that sometimes includes implicit learning [4]. In any case, in implicit memory tasks, acquisition is often tested on exemplars from the acquisition set, as in perceptual priming or repetition priming, while implicit learning more often use novel stimuli created according to the same principles as the acquisition or training set [4]. Generally, we will not review priming studies here, but we will include one implicit memory task [27] since it fulfills the criteria for implicit learning and does not use simple exemplar recognition but rather generation with the instruction to exclude the acquisition set.

The aim of this review is to provide an overview of research in (healthy) human subjects - but animal data will be reported when it adds insight into the mechanism(s) demonstrated in humans. In animal studies, tasks are sometimes conceptualized as procedural learning but there seems to be some confusion over what procedural or implicit learning should mean in animal models. However, there are recent examples of rule learning in cotton-top tamarins [28, 29], songbirds [30, 31], and rats [32] that are relevant for future neuropharmacological studies of implicit learning in animals.

Clinical data is considered where there is lack of other data and when this provides insights beyond clinical relevance. We also note that related reviews have covered the neuropharmacology of cognition and memory in general [33], neuropharmacology of working memory [34], the role of basal ganglia, including its neuropharmacology, in habit formation [35-37], the role of neuropeptides, especially vasopressin, in animal studies of learning and memory [38] and the role of dopamine in actions and habit formation [39]. But, there is yet no systematic review with a focus on the neuropharmacology of implicit learning.

In this review, one might sometimes get the impression that we are suggesting that there is a single effect or effect direction of a pharmacological agent acting on a transmitter system, for example. Generally, this should be understood as a (over-)simplification of the true, underlying complexity which is more closely related to for example plasma or tissue concentrations of the pharmacological agent, as well as its pharmacokinetics and pharmacodynamics. In this respect, studies reporting dose-response-curves are more informative than studies which only investigate a single dose level. However, the former type of study has, to a large extent, not yet been conducted in the context of implicit or procedural learning. The ideal would be to discuss investigations of moderate increases or decreases in neurotransmitter function, coupled to moderate increases or decreases in plasma concentration of agonistic or antagonistic agents, within a physiologically relevant range (as distinguished from a wider pharmacological range). For the purpose of this review, we tentatively assume that the studies reviewed are more or less appropriately designed from this point of view, as long as no conflicting information is reported in the literature. However, it has to be kept in mind that full-range dose-response studies are generally lacking.

## 2. A BRIEF OVERVIEW OF RELEVANT NEUROANATOMY

**Fig. (1):**
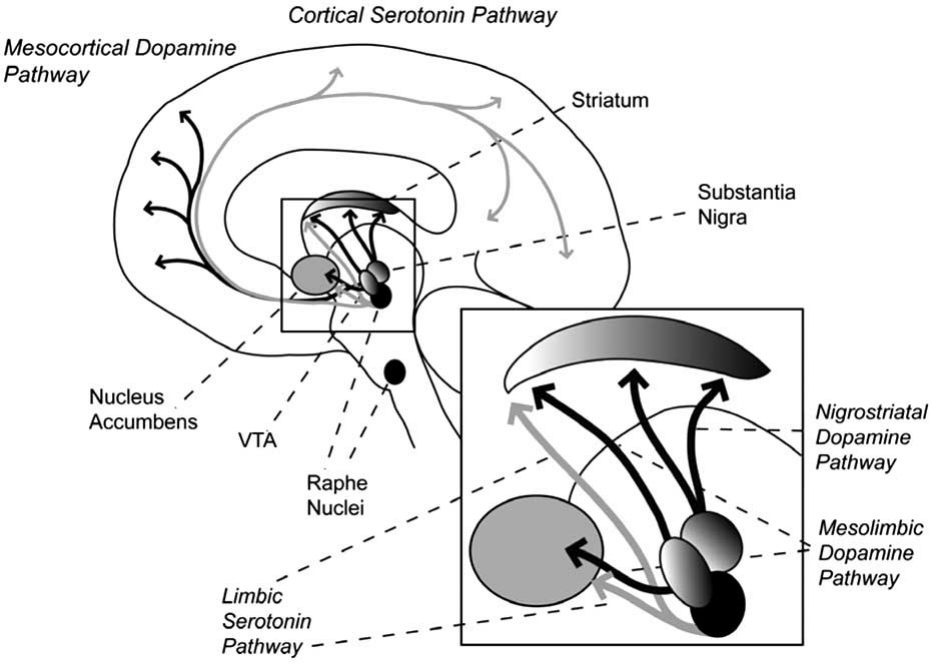
Dopamine pathways are drawn in black and serotonin pathways in grey. The dopamine system has a substantial overlap with fronto-striatal circuits known to play a key role in implicit learning. The serotonin system is also overlapping with these circuits to a large degree.

Here we provide a very brief overview of the neuroanatomy and the clinical relevance of the four main neuromodulators discussed in this review. The dopamine (DA) system is divided into four major pathways (Fig. **1**). The mesolimbic and mesocortical system originate in the ventral tegmental area (VTA) and project to limbic (nucleus accumbens/ventral striatum) and prefrontal cortex, respectively. Over-activity in these systems is related to various psychological or psychiatric symptoms, including euphoria, psychosis, and schizophrenia. The nigrostriatal DA system originates in substantia nigra of the mesencephalon. The pathway projects primarily to the dorsal striatum, and under-activity is associated with Parkinson’s disease (the tubero-infudibular DA system is not considered here).

**Fig. (2):**
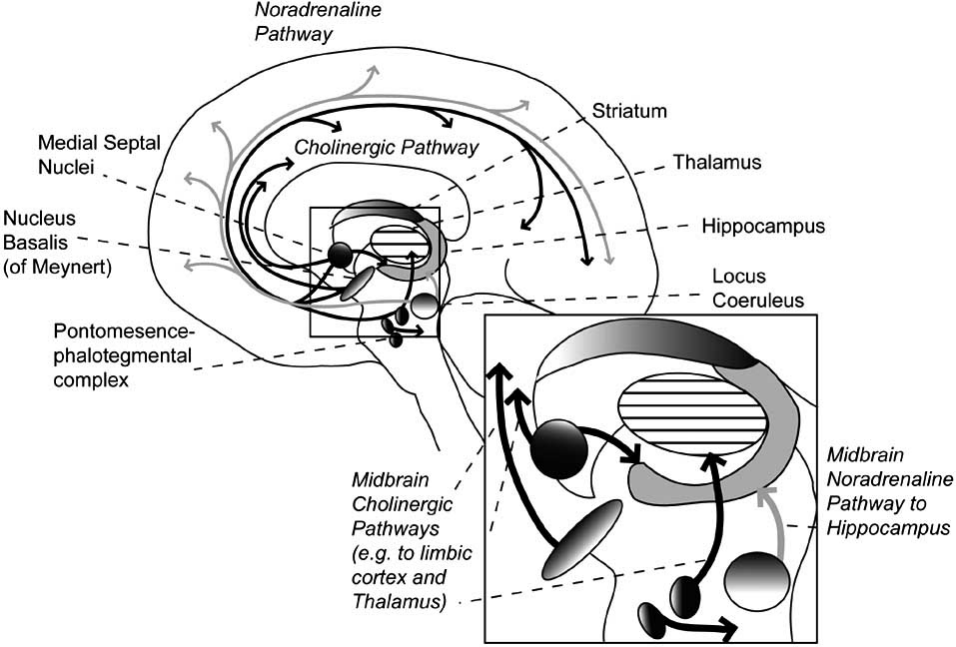
Noradrenaline pathways are drawn in grey and acetylcholine (cholinergic) pathways in black. They project directly to hippocampus but not to striatum. More generally, these two systems are not projecting as specifically to fronto-striatal circuits as the dopamine and serotonin system.

In addition to VTA and substantia nigra, the monoaminergic brainstem consists of the raphe nucleus and the locus coeruleus, from which the serotoninergic (Fig. **1**) and the noradrenergic systems (Fig. **2**) originate, respectively. The noradrenergic system projects to the cerebellum, hippocampus and neocortex while the serotonin system projects to all of these structures as well as the striatum. The nucleus basalis (of Meynert) in the ventral forebrain is the origin of cholinergic neurons projecting to cortex and limbic structures. The medial septal nuclei provide cholinergic projections to the cortex, limbic structures, and the hippocampus (Fig. **2**). The third cholinergic origin is the ponto-mesencephalo-tegmental complex (laterodorsal tegmental and pedunculo-pontine tegmental nuclei) which projects to the brainstem, thalamus and the basal forebrain. Cholinergic under-activity is associated with Alzheimer’s disease. In the context of this review, it is notable that neither the noradrenergic nor the cholinergic system projects directly to the striatum.

The most well-studied interactions between the systems briefly outlined are: (1) serotonin dependent dopamine release; (2) dopamine and serotonin affecting acetylcholine release in the basal forebrain; and (3) interaction between dopamine and acetylcholine in the striatum. A dopamine 1 (D1) receptor activation depolarizes cholinergic interneurons and enhances acetylcholine release. In contrast, activation of dopamine 2 (D2) receptors on cholinergic interneurons inhibits striatal acetylcholine release, reviewed in [40]. There is also much evidence for serotonin agonists increasing acetylcholine release in the basal forebrain [41]. Serotonin can also modulate dopamine function and dopamine release both positively and negatively (primarily by modulating dopamine levels in cortex). But, there is also evidence that DA neurons in the VTA are under excitatory control by serotoninergic neurons in prefrontal regions, reviewed in [42]. Thus, there are several possibilities for complex interactions between these systems, which are yet to be described. Such interactions cannot be excluded as the actual cause of behavioral effects that we here interpret as findings supporting the involvement of a particular transmitter system in implicit or procedural learning. This is a difficulty that can only be resolved through the systematic study of multiple systems simultaneously, an important topic for future research.

## 3. RELATED MODELS APPLIED TO IMPLICIT LEARNING

We start with reviewing five computational models that focus on the basal ganglia and cover relevant aspects related to the neuropharmacology of implicit learning: (1) Doya’s computational model of reinforcement learning; (2) Berns and Sejnowskij’s model of sequence production; (3) Frank’s model of probabilistic learning and; (4) Ashby, Ennis and Spiering’s COVIS model of perceptual categorization; and (5) Ashby, Ennis and Spiering’s SPEED model of automaticity in perceptual categorization [43-46].

Reinforcement learning is a computational framework concerned with how an agent ought to take actions in an environment in order to maximize its reward. The agent is dependent on getting a variable reward signal in order to learn how to maximize its reward and in this sense, the learning is supervised. Implicit learning on the other hand typically does not include supervised feedback. Thus, we will now comment on why we review Doya’s model for reinforcement learning [46] in the context of implicit learning. Petersson and colleagues [47, 48] described a non-supervised acquisition mechanism based on predictive learning that provides system internal feedback based on internal prediction. Thus, despite definitional differences between non-supervised implicit learning and reinforcement learning, we think they might partly overlap in their mechanistic implementation. Common mechanisms might include computation of predicted outcomes and the presence of (internal) error signals. A control signal that determines the amount of exploration of unknown territory versus exploitation of already acquired knowledge is a part of the reinforcement learning framework that could also be related to implicit learning. Such a control signal would be relevant as an interface between the overall goals of the organism and the core learning mechanism in both implicit and reinforcement learning. From this point of view, early phases of implicit learning might for instance be characterized by exploration rather than exploitation (i.e. goal driven action execution). Common mechanisms would probably be implemented in the basal ganglia, a neuroanatomical focus for both implicit and reinforcement learning.

The functional roles of neuromodulators have been conceptualized in terms of control parameters regulating learning mechanisms. The characteristics of such control parameters were specified by Doya [46] for reinforcement learning, based on evidence related to the basal ganglia learning system. Doya [46] proposed the following roles of the monoaminergic and cholinergic systems: (1) dopamine controls a global learning signal that encodes the discrepancy between predicted and received reward; (2) acetylcholine controls a learning parameter scaling this signal; (3) serotonin controls a discount factor, assigning exponentially less weight to reward predictions further in the future; and (4) noradrenaline controls a noise term in the action choice that is supposed to balance exploration and exploitation. In the case of dopamine, the model is uncontroversial and in line with the other models reviewed in this paper. 

The perspective on serotonin is also uncontroversial and based on evidence that low levels of serotonin are associated with decreased behavioral inhibition and impulsiveness, for example prioritizing small immediate rewards before larger, delayed rewards (i.e., promoting “greediness”). In a recent review [49] this view was supported and serotonin was again considered as important for delay discounting and perhaps also for uncertainty discounting (meaning that less weight is assigned to uncertain predictions). The suggested role of noradrenalin in Doya’s model has been supported by primate perceptual data, for example visual discrimination [50]. However, it is an open question whether its role as a controller of the balance between exploration and execution generalizes to higher cognitive functions.

The reason given for modeling acetylcholine as controlling the learning rate (i.e., rate of acquisition) was that acetylcholine (ACh) modulates long-term potentiation (LTP) in various learning tasks through muscarinic ACh receptors. However, LTP is also dependent on the AMPA and N-methyl-D-aspartic acid (NMDA) receptors, and is modulated by dopamine, so this part of the model is probably too simple. From the perspective of this model, it can be predicted that implicit and other types of learning would be facilitated, at least in the acquisition phase, by moderate dopamine increases (since a magnified learning signal could translate into improved acquisition). Somewhat higher than normal levels of acetylcholine during initial acquisition could facilitate fast learning, but it could also lead to an unstable oscillatory solution to the acquisition problem. Low learning rates means proceeding accurately but very slowly across the error surface. Thus, for implicit learning over very long periods where high accuracy is crucial, low acetylcholine levels could be preferred. In any case, performance impairment is expected in case of dopaminergic or cholinergic dysfunction.

Lower levels of dopamine and acetylcholine after acquisition might prolong the time window during which the acquired knowledge is remembered. Too high levels, both during the acquisition and the test phase, would mean that what is learnt might be overwritten and this would translate into instability and lower performance of the learning system. Implicit learning is predicted to improve by moderately high serotonin levels that optimize long-term prediction of reward and perhaps by somewhat lower than normal levels of noradrenalin (optimizing exploration), at least during acquisition.

One suggestion in Doya’s model was that VTA DA innervation of the prefrontal cortex mediates the selection of which task-dependent representation of states and actions (i.e., the input to the striatal reinforcement learning equations) should be acquired. For instance, there is always a choice at what level of detail an environmental object should be internally represented and the level of detail chosen might substantially affect the acquisition process. Thus, dopamine is not only signaling discrepancy between predicted reward and actual reward, but it may also be a key player in selecting the input on which predictions are computed. Ellis & Nathan [34] suggested that a mechanism for this function could for instance be dopamine mediated control of working memory [for a review of the pharmacology of working memory see 34]. This was based on evidence that sustained prefrontal activity during working memory tasks is dependent on the amount and type of reward associated with the memorized items. Studies using dual task paradigms have showed that working memory is important for implicit learning of hierarchical relations, such as learning the positional dependencies between elements in a sequence in AGL [for a review see 4]. Two studies have demonstrated a correlation between AGL classification performance and working memory in schizophrenic patients [51] and healthy volunteers [52].

Berns and Sejnowskij [44] modeled the effect of dopamine as the difference between predicted and actual reward similarly to Doya [46]. In their model of sequence production, sequences are generated by the basal ganglia. A short term memory loop of inhibitory recurrent connections between globus pallidus externa and the subthalamic nucleus plays a central role in this model. This memory device, proposed to operate on a timescale of 10 to 100 ms, can store sequences of states projected to globus pallidus externa from striatum. Action selection is made through a comparison of the input to globus pallidus interna by lateral connections from globus pallidus externa and direct projections from striatum, in this model. The recurrent circuit between globus pallidus externa and the subthalamic nucleus was also considered in Doya’s model [46], where it computed the global DA learning signal. Berns and Sejnowskij [44] further suggested that dopaminergic neurons in substantia nigra and ventral tegmental area fire as a consequence of both external and internal reward. The tacit assumption here is that a tonic level of dopamine activity is required to maintain synaptic efficacy and therefore allow the learning signal to be either positive or negative [this also holds for 46]. It was also suggested that dopamine modulates the long-term potentiation/long-term depression (LTP/LTD) of both striatal and pallidal synapses by altering the concentration of intracellular calcium.

Franks’s model [45] describes the role of dopamine in the basal ganglia and prefrontal cortex in probabilistic learning; for example, the weather prediction task [53] and probabilistic reversal learning [45]. These tasks were chosen for the purpose of modeling behavior of medicated and non-medicated Parkinson patients, since these patients seem to be impaired on tasks that rely on trial-and-error learning. The author emphasized that a large dynamic range in dopamine release is critical for this kind of basal ganglia dependent learning. Unfortunately, no direct comparison was made between the role of dopamine in trial-and-error dependent and independent implicit learning in the model. Bearing Frank’s model in mind, it is of particular interest for the current review to establish whether dopamine has been implicated in implicit learning that does not rely on trial-and-error learning (i.e. tasks not using feedback). Frank reviewed empirical evidence that positive and negative feedback has opposite effects on the dopamine release, in line with Doya [46] and Berns & Sejnowski [44]. As in Berns & Sejnowski [44], it was suggested that the convergence of the indirect and direct pathway in the globus pallidus interna indicates that a competitive mechanism might control the output to the thalamus.

Ashby and colleagues’ COVIS model and its extension SPEED are neurobiological models centered on category learning. In their sense, category learning is based on information integration as opposed to explicit rule application. This makes the models interesting from the point of view of implicit learning [43]. The model assumes that an implicit basal ganglia system uses procedural learning mechanisms to acquire categories. It proposes a cellular mechanism for this, in the form of dopamine mediated synaptic strengthening, connecting cortical axons with medium spiny dendrites in the striatum, with a focus in the body of the caudate nucleus. As in Berns & Sejnowski [44], Frank [45], and Doya [46] dopamine was regarded as a learning signal for unexpected reward or omission of expected reward in both striatum and the prefrontal cortex. SPEED adds a neural mechanism for automaticity to COVIS. This mechanism strengthens cortico-cortical pathways through Hebbian learning. Ashby *et al*. argued that this mechanism is relatively independent of dopamine, based on the fact that dopamine signals in prefrontal cortex have poor temporal resolution. This is in line with the prediction we have outlined above, building on Doya’s model [46]: an intact dopamine system is important in the early phase of learning, but perhaps less important later in the acquisition process, where e.g. skills become more automatic. It is also in line with the reasoning in the review of Wickens *et al*. [39] where pharmacological disturbances in early stages of learning are thought to impair performance on habit learning tasks generally by disrupting the throughput of information across task-relevant cortico-striatal synapses. Such impaired performance was shown for acquired appetitive responses in rats [39].

Altogether these models converge in their view on striatal dopamine as a global learning signal playing an important role in reinforcement, sequence, category, and probabilistic learning. Prefrontal dopamine was suggested to be involved in the selection of acquisition of internal representations of the environment, which form the input to the reinforcement learning processes in the striatum [46]. It was believed that dopamine determines the strength of cortico-striatal synapses, but that it is of relatively minor importance for cortico-cortical pathways. These cortico-striatal pathways are suggested to play a role in the development of automaticity in a gradual process. Automatic processing will eventually rely on cortico-cortical pathways only. This suggestion is similar to how memories are thought to iteratively consolidate in cortical areas after first having been dependent on hippocampus as a hub binding together distributed storage sites in neocortex [23, 43, 54, 55]. The time course suggested, including an initial important role of dopamine which later fades out, can be interpreted as consistent with the time course in Doya’s model [46]. Dopamine might be particularly important for implicit learning involving trial-and-error with feedback [45]. To sum up the model’s view on the other neuromodulator systems, acetylcholine is seen as controlling the balance between memory vs. renewal; serotonin is controlling the balance between short and long-term prediction of reward; and noradrenaline controls the balance between exploration and exploitation.

## 4. DOPAMINE

Nagy and colleagues [56] measured plasma levels of homovanillic acid (HVA) as a marker of dopamine metabolism in healthy volunteers during the chaining task, which is a simple sequence learning task with feedback. This task was constructed so that errors are made if subjects respond in a stimulus-response fashion, without having acquired the exact positions of the elements in relatively short sequences (maximum 4 items long). In a test phase without feedback, performance was impaired in amnesic patients, but not in Parkinson’s patients, while the opposite pattern was seen in the training phase [56]. This evidence suggests that there might be an implicit learning component in the training phase that is related to dopamine function, since the nigrostriatal dopamine pathway is malfunctioning in Parkinson’s disease. Nagy and colleagues [56] reported that increased dopamine metabolism (prior to acquisition) was negatively correlated with the number of errors. No significant correlations were found between plasma indicators of serotonin and noradrenaline metabolism and the number of errors. Interestingly, there was no correlation between dopamine metabolism and error rates in the later test phase, where the subject is supposed to apply the acquired sequence knowledge. This supports the hypothesis that dopamine is more relevant in an early learning phase, a possibility we have discussed in the section on models related to implicit learning above. An alternative explanation is that performance is only dopamine dependent during feedback, which was only present during the training phase.

A number of studies that have investigated how learning is affected by antipsychotic treatment of schizophrenia are interesting also from a non-clinical perspective, since they point to an involvement of the dopamine system in implicit learning independent of the disease [for a recent review of implicit sequence learning in schizophrenia which also discusses clinical aspects, see 57]. In a study that compared the new antipsychotic olanzapine with the traditional benchmark drug haloperidol and equivalents, it was concluded that implicit learning in the serial reaction time task [15, 18] was not impaired in non-treated schizophrenia (although this was not directly tested). Classical neuroleptics like haloperidol did impair implicit learning in the SRTT, while this was not the case for olanzapine. No detrimental effects were found in schizophrenic individuals under treatment with olanzapine compared to untreated healthy controls. This between drug effect was specific for implicit learning in the serial reaction time task (measured as the relative improvement in reaction times over blocks) since no such effect could be demonstrated in an explicit spatial memory task [58].

The pharmacological profile of olanzapine is complex and involves different actions on the DA, serotonin (5-HT) and cholinergic systems. However, some aspects of the compounds complex effect have been studied. In schizophrenics treated with either olanzapine or haloperidol, dose dependent effects of D1 and D2 availability in the striatum measured by SPECT has been demonstrated [59]. Haloperidol is a relatively pure D2-antagonist with higher affinity for the D2-receptor than olanzapine. A tracking task was performed directly after injection of a radioligand with high D2-specificity. Performance correlated significantly with D2-binding in the striatum in a haloperiodal treatment group, but not in a olanzapine group. Interestingly, and consistent with the data reported above, this study [59] also reports impaired procedural learning under haloperidol treatment. The performance of patients treated with olanzapine was on the other hand not significantly different from that of unmedicated healthy controls. These findings are in line with findings by Purdon *et al.* [60], where haloperidol caused impaired procedural learning in the Tower of Toronto task, a puzzle of intermediate difficulty compared to the Tower of London and Tower of Hanoi. 

In all of these Tower tasks, there are disks stacked in ascending order of size on one rod, the smallest disk at the top. The objective of the task is to move the entire stack of disks to one of two other rods, moving one disk at a time and without placing a bigger disk on top of a smaller disk. In simple versions, the solution with the least number of moves can be found by problem solving, at least in healthy subjects. In harder versions, the procedural learning component becomes more prominent and this goes along with an increased the number of moves that varies depending on procedural learning proficiency. The Toronto version of the Tower tasks was designed to maximize the procedural learning component, in the sense that individual moves should not be memorized and subjects should be unable to consciously recall a successful sequence [21]. The impaired performance on the Tower of Toronto task in schizophrenic patients was found after six months of medication [60]. This result suggest that D2 blockade has a general negative impact on this procedural learning task, but that this impact is circumvented by other aspects of the newer drugs like olanzapine. The authors suggest that either the anticholinergic properties of olanzapine or the increased 5-HT_2_ receptor blockade might compensate for the D2 receptor blockade. A stratified analysis showed trends for better performance by patients who had gotten additional anticholinergic treatment compared to those who had not. The fact that these effects are found in striatum [59] and perhaps the dorsal striatum specifically is interesting from an implicit learning perspective, since striatum is one of the structures most consistently activated by implicit learning tasks [2]. Altogether, these three studies provide some evidence for dopamine involvement in implicit learning independent of trial-by-trial feedback, since no feedback was provided [58-60].

Kumari and colleagues [61] studied behavioral performance in healthy subjects in a procedural learning task where subjects had to touch one of four locations of a target that was moving with random or predictable movements, with no training prior to drug administration. This task resembles versions of the serial reaction time task [15, 18]. Haloperidol and d-amphetamine (a dopamine agonist) had opposite effects. D-amphetamine shortened the response times of predictable movements compared to random movements, whereas in the haloperidol group, they were stable or even increased. These results are consistent with Doya’s model (i.e., the effects go in the predicted direction). However, the interpretability of the d-amphetamine part of the results is somewhat weakened by the fact that d-amphetamine acts on many systems and may speed up responses in general. This criticism is mitigated by the fact that the effect of faster response times was measured as the difference between the reoccurring sequence and the random sequences and a general speed up would mean that this difference would have stayed unaffected.

Two studies in rats used procedural learning paradigms to assess effects of D1 and D2 receptor antagonists as well as long term effects of striatal dopamine depletion on sequential motor learning. If the dopamine depletion exceeded 40%, sequential motor learning was impaired up to six weeks after the depletion treatment [62]. Injection of both D1 and D2 receptor antagonists had detrimental effects on a task supposed to correspond to the human serial reaction time task. The rats had been trained on this task for 40 days prior to the 4 day treatment period. Response rate and accuracy was lower and reaction times were longer after excessive dopamine depletion. In a between subjects comparison, it was found that reaction times were specifically prolonged in relation to manipulation of the D2 receptor [63]. A possible explanation for D2 specificity is that D2 receptors inhibit acetylcholine release, while D1 receptors have the opposite effect. Such complex interaction between these two systems calls for further research. A possible D2 specificity for dopaminergic modulation of implicit learning should also be further investigated and we note that the results on haloperidol above are consistent with the D2 specificity hypothesis.

Two studies reported negative results. Czernecki and colleagues [64] concluded that L-dopa (a precursor to dopamine) treatment in patients with Parkinson’s disease did not ameliorate impaired implicit learning in Parkinson patients. The authors argued that the gambling task used in the study is an implicit learning task since both patients and controls showed progressive learning in absence of explicit awareness. The task was to distinguishing which two decks of cards out of four were advantageous in the long run (i.e., producing larger monetary reward, later) and which two were disadvantageous (smaller monetary reward, sooner). The explicit component was assessed after the task and 50% of the controls and 13% of the patients became aware of the distinction between the decks. This task has clear similarities to the classical 2-choice probabilistic learning task in the sense that feedback on the subject’s decisions is provided. The authors wrote “This kind of implicit learning would require processes of long-term consolidation which are probably poorly sensitive to the short-term fluctuations of dopaminergic therapy” [64].

However, when considering the data presented in this study, it looks like the L-dopa administration had a detrimental effect on learning in the gambling task. In a session with 5 blocks, controls and patients without treatment showed highly significant increases in performance, while patients with treatment showed no significant improvement. This pattern of results was not present in a second session, administered the next day. The patients who were non-treated earlier received L-dopa and the treated patients received no L-dopa. Both patient groups showed stable performance over blocks, in contrast to the controls who were still improving significantly. There are several possible reasons for the absence of learning in the patient groups in the second session (for instance a motivational drop), but the data is also consistent with the interpretation that L-dopa treatment impairs further learning in this study.

Witt and colleagues [25] compared four patient groups with controls in an artificial grammar learning task. The purpose was to test the causal role of the basal ganglia in this task through measuring implicit learning in advanced and non-advanced patients. The advanced patients were measured both in a medicated stage (varying treatments but all with positive effects of L-dopa treatment) and after at least 12 h of withdrawal from all drugs. The role of cerebellum was also investigated on patients with cerebellar degeneration. No significant effects were found between patient groups and controls or between treatments. There was a tendency for Parkinson patients being more affected the more advanced the disease was and thus the possibility that the absence of results is potentially an effect of limited statistical sensitivity is important to note [25].

Altogether, these results suggest that intact dopaminergic function is necessary for normal performance on implicit learning tasks, most clearly in the acquisition phase, as predicted [39, 43-46]. There is evidence for improved learning when moderate levels of dopamine agonistic agents are administered. There is even more evidence for impaired learning by antagonistic agents. The effects show anatomical convergence in the striatum, most prominently the dorsal part [58-61]. Taken together, these results support the hypothesis that dopamine determines the strength of cortico-striatal synapses during the development of automaticity. It has been demonstrated that individual differences in the state of dopamine metabolism during learning affect the acquisition. There is also support for a role of the dopamine system in tasks that do not rely on trial-and-error feedback learning [58-61]. For a review on all dopamine results, see Fig. (**3**), Table **1**.

## 5. SEROTONIN

Citalopram, a selective serotonin reuptake inhibitor (SSRI), increases immediate sensitivity to misleading feedback. Chamberlain and colleagues [65] investigated citalopram in the context of a two alternative forced choice probabilistic learning task. There was a 8:2 ratio of positive:negative feedback for the correct choice and the converse for the incorrect choice. Without prior training on the task, performance was impaired after oral intake of a single dose of the selective serotonin reuptake inhibitor (SSRI) compared to placebo controls, both in terms of response times and accuracy. This could be due to either sub- or supra-optimal levels of serotonin, both possible consequences of initial SSRI administration. SSRI was used to increase serotonin levels but initially it can have the opposite effect. Whether the observed effect was due to too low or too high serotonin levels is impossible to say, but it seems clear that the placebo controls’ levels are closer to optimal.

Cruz-Morales and colleagues [71] exposed rats to restraint and 24 h later these were exposed to an elevated maze stressor which had open and closed arms. The authors suggested that procedural learning in this task depends on serotonergic activity in the striatum [71]. We note that increased avoidance latencies in elevated mazes has also been observed after 3,4-methylenedioxymethamphetamine (MDMA) exposure, which among other effects causes serotonin depletion. The increased avoidance latencies were measured three months after 48 h MDMA exposure. The training had begun directly after the exposure [72]. In a similar study, the same effect was observed during three weeks of behavioral tests after a single injection of MDMA [73]. However, in both cases, the effects were interpreted as resulting from increased anxiety and the procedural learning perspective was not taken. These three rat studies suggest a role of serotonin in avoidance learning, which is the main learning component in the elevated maze task (see Fig. (**4**), Table **2**). Avoidance learning in the elevated maze task has been interpreted as a procedural learning paradigm in at least one study [71], but its relevance for implicit learning is still controversial [for a discussion of the relevance of maze task for implicit learning, see 19].

Depletion of the serotonin precursor tryptophan impaired response speed in probabilistic reversal learning in healthy volunteers [67]. The task used was a classical probabilistic learning task with feedback, repeated in two sessions after intake of an amino acid mixture with or without tryptophan. The ratio 8:2 of positive:negative feedback given in the trial by trial feedback was inverted once during the session. Tryptophan depletion, a well-established method for reducing serotonin levels in the brain, increased response times, but only during the first session when the task was new to the subjects. This is consistent with the predictions from Doya’s model, where intact serotonin was predicted to affect implicit learning early more than late in the process. In an almost identical design, it was shown that probabilistic reversal learning during tryptophan depletion elicits a task related increase in the blood oxygen level dependent (BOLD) response (functional magnetic resonance imaging, FMRI) of the dorsomedial prefrontal cortex. A marginally significant trend for increased response latencies related to the lower serotonin levels was also found [66]. Moreover, the dorsomedial prefrontal cortex has been reported in FMRI studies of implicit artificial grammar learning without performance feedback [5, 8] as well as explicit artificial grammar learning with performance feedback [74-77]. These are some indications of a relationship between the serotonin system, the dorsomedial prefrontal cortex and implicit learning with and possibly without feedback, but further research is needed on this topic.

Taken together, these results provide some evidence for a serotonergic involvement in procedural learning, in particular in probabilistic learning tasks, in line with the predictions of Cardinal [49] and Doya [46] (see Fig. (**3**), Table **1**). However, a serotonergic involvement in a classical human implicit learning task like SRTT or AGL with and without feedback remains to be demonstrated.

## 6. NORADRENALINE

There are yet no positive results showing a role for noradrenaline in implicit learning. Chamberlain *et al*. [65] found effects of serotonin on probabilistic learning but did not find any significant changes on the same task after intake of the selective noradrenaline reuptake inhibitor atomoxetine. In addition, reboxetine, another selective noradrenaline reuptake inhibitor, showed no effect on finger sequence learning [80]. In a study that used clonidine, which among other things reduce the noradrenaline turnover, no effect was observed on a procedural motor learning task described below [68]. Altogether, the absence of a relation between noradrenaline and implicit or procedural learning suggests that if such a relation exists, it is not prominent compared to other neurotransmitters, like dopamine for example.

## 7. ACETYLCHOLINE

Frith and colleagues [68] found an effect of the acetylcholine antagonist scopolamine in a procedural motor learning task where subjects had to learn to move a joystick in a mirror reversed manner to track a slowly moving target. After brief training on the task, subjects had intravenous infusion of scopolamine and about half an hour later, the proficiency in this task increased significantly. The comparison was made between the scopolamine group and clonidine or placebo groups. Comparing explicit and implicit processing, Nissen and colleagues [81] found effects of scopolamine on explicit but not implicit motor learning, subsequently replicated by Bishop *et al*. [82]. This type of experimental design only provides a simple dissociation and leaves open whether the assessment of implicit processing was sensitive enough to detect behavioral changes or not. 

Wenk and colleagues [79] found impaired choice accuracy in rats after lesions to the basal forebrain cholinergic system and the raphe nucleus, but not the locus coeruleus, in the probe phase of a T-maze task after at least four days of acquisition prior to operation. However, these results involve a spatial dimension that might be affected rather than the procedural learning component. Roloff *et al*. [83] demonstrated a dissociation between these two components and they showed that both the spatial and the procedural learning component are affected by muscarinic acetylcholine receptor signaling in a series of four experiments. Vanderwolf [78] found impaired acquisition in a shock avoidance test after administration of scopolamine before acquisition and on retention, compared to another group of placebo treated rats. In a swim-to-platform test scopolamine only impaired acquisition but not retention [78]. The relation between these rat tasks and implicit learning in humans is not straight forward to interpret [19] but at least some authors think that the role of acetylcholine in procedural learning in rats is uncontroversial and well-established [83]. In summary, there is not enough evidence to support a role for the cholinergic system in implicit learning generally, although there is evidence for impaired procedural learning in rats and some mixed results in humans.

## 8. GABA AND BENZODIAZEPINES

In an interesting placebo controlled study the MTL memory system, including the hippocampus, was deactivated by using a GABA-A receptor agonist (midazolam; GABA-A receptors being densely expressed in the hippocampus). This resulted in improved implicit transitive inference, defined as the ability to infer transitive relations. A simple example of such a relation is the following: from A > B and B > C, it follows that A > C. The transitive relations used in the study were more complex. Interestingly, an explicit name recall task was impaired after the same treatment [69].

However, Greene [84] argued that there was no convincing evidence that the hippocampal inactivation caused the behavioral findings in [69]. We agree that other causes cannot be excluded although the hippocampal inactivation account is certainly consistent with other experimental evidence. In a reply to Greene, Frank *et al*. [85] emphasized that there is substantial evidence for inactivation of hippocampus after intake of midazolam, while there is no evidence for deactivation of for example striatum under the same conditions. Frank *et al*. [69] interpret their results as at least partly resulting from a shifting balance in a competitive relation between striatum and hippocampus. Such a competitive relation is supported in a recent study of implicit learning using the artificial grammar learning task. The study shows medial temporal lobe deactivations during artificial syntactic processing [12].

Cooperative interactions between the basal ganglia and the medial temporal lobe have also been reported [86]. However, other neocortical causes of the behavioral effects cannot be ruled out. We also note that a dissociation of memory performance and the sedative effects of midazolam have been replicated using concentrations of midazolam which caused severe amnesic effects and equi-sedative doses of the drug fentanyl. Fentanyl produced no effect on recognition of words from a wordlist and verbal recall of pictures [87]. Thus we conclude that the results of Frank *et al*. [69] provide some evidence of a role for the GABA-A receptor in an implicit learning task. In this task subjects were instructed to base their decisions on their “gut feeling” and subjects generalized by applying previously acquired regularities to novel items. These are two important features of how the AGL task is commonly administered and they make this version of the implicit transitive inference task more interesting from an implicit learning perspective. The results might be due to a deactivation of hippocampus subsequent to midazolam intake. Alternative explanations are: (1) increased activation of the indirect pathway of the striatum, which has been shown after administration of midazolam, measured with two different neuropeptide markers [88, 89] (2) increased dopamine and dopamine metabolite levels as well as turnover rate in the striatum, which has been demonstrated after administration of a benzodiazepine [90] and (3) neocortical effects.

However, modulation of GABA receptors with benzodiazepines has also resulted in opposite effects. Placebo controlled pentobarbital impaired procedural learning in a sequence learning task resembling the serial reaction time task [70]. Lorazepam induced impairments in both explicit and implicit learning in a word stem completion task where implicit learning was defined as the rate of erroneously included word stems from a word list [27]. This is an example of an implicit memory or priming task, which might or might not be related to implicit learning. Altogether, these findings suggest that the GABA-A receptor system is involved in implicit or procedural learning. Some concerns about specificity remain since benzodiazepines also induce altered states of wakefulness and attention. Since neither plasma levels or dose-response curves were reported, it is possible that the opposite direction of the results (see Fig (**3**), Table **1**) might be a dose effect, for instance too high GABA levels in the last two studies above (although no specific details in the administration or dose of the drug support this suggestion).

## 9. AMPAKINES 

Ampakines have theoretically interesting properties and effects, including induction of enhanced plasticity and regional specificity of induction as well as amplification of LTP. We review this class of drugs although the potential effects of ampakines on implicit learning have not yet been investigated. Ampakines are allosteric modulators of the AMPA receptor that partially interrupt desensitization and deactivation of the excitatory postsynaptic current, producing a net effect of increased glutamate transmission, both in terms of amplitude and duration of the excitatory postsynaptic current. The NMDA glutamate receptors that trigger LTP are both voltage-gated and ligand-gated. Mg^2+^ is blocking the ion channel. Co-localized AMPA receptors can cause a rapid depolarization that removes the magnesium ion and lets calcium flow into the cell. Because the NMDA channels are relatively slow to open, this NMDA dependency on AMPA postsynaptic receptor-mediated currents will depend on both the duration and the amplitude of the currents [91]. Thus, ampakines can have large effects on these NMDA channels. In terms of LTP, the induction threshold is lowered and the magnitude of the LTP increases [92]. An additional, secondary effect is increased production of brain derived neurotrophic factor (BDNF), in turn enhancing plasticity [92]. The mechanism of ampakines is neither agonistic nor antagonistic, but a dynamic modulatory effect on stimulus driven, endogenously produced, excitatory transmitter release. These properties might be especially suited for improving implicit learning. Different positive modulators (potentiators) of AMPA receptors have been developed. The ampakine CX516 affected deactivation selectively, while benzothiazide and cyclothiazide affected desensitization selectively, and CX554 affected both [for reviews see 92-94]. However, there are no studies that report administration of the later two types in humans.

CX516 has been tested in healthy young adults at fairly high doses [95]. In a placebo-controlled study, Ingvar and colleagues [95] found improved performance on a spatial maze task and odor recognition, pictorial associations and another simple association task after ampakine administration. All these tasks are dependent on long-term memory processes in the different (spatial, odor, visual) domains. The design was a within subjects design were the test battery was repeatedly administered for five test days, two after 300 mg CX516 ampakine administration and three after placebo. In another study, healthy 50-65 year old subjects showed improved recall of syllables [96]. Goff and colleagues [97] tested 19 schizophrenic patients and found therapeutic effects of an increasing dose of at least 300 mg and maximally 1200 mg CX516 a day, during four weeks. Improvements of attention and memory were seen, measured with test such as Wisconsin card sorting test, letter-number span, verbal learning test, fluency tests and trail making tests. However, these results were not reproduced in a larger sample (n=105) where patients were given a 300 mg dose a day [98].

CX717 is another ampakine which have been tested at high dosage in humans during electroencephalography (EEG) assessment. Modification of EEG activity during sleep in all bands except the theta band was demonstrated [99]. The authors interpreted the pattern of changes in oscillatory activity as increased arousal [99]. However, in a follow-up study, similar dosages were not found to affect behavior by night shift workers performing a delayed match-to-sample task, a vigilance psychomotor task, and a wakefulness test. This test was done during four nights where a different dose of CX717 was administered each day [100].

Interestingly, there was a dose-dependent effect of the same ampakine on a delayed match-to-sample task in rhesus monkeys. This was true for both normal and sleep deprived monkeys in a within subjects design [101]. The delayed match-to-sample task is a working memory task where a choice between two items has to be made, so that the chosen item matches some feature of a target item that has been kept in mind during a delay period. This task has been extensively used in animals and for cross-species comparisons [102]. The enhancement of performance in the delayed match-to-sample task due to ampakine administration was specific to the brain regions involved in the task as measured by glucose consumption. This was assessed with PET [101].

Faramptor is an ampakine developed for improvement of negative symptoms and cognitive function in schizophrenics [103]. It is supposedly more potent than CX516 [103]. These authors tested healthy elderly volunteers and found effects of a single administration of 500 mg faramptor on accuracy of incidental learning in symbol recall and recognition. There were no effects on word list recall. Recall here refers to a memory task where the remembered item has to be produced (e.g. in speech, writing or drawing). Indication of which items are the remembered ones is sufficient in recognition tests.

In summary, ampakines have been tested in humans but mainly for clinical purposes and mainly for declarative memory and working memory. These studies provide evidence that ampakines can improve memory and learning in human subjects. In addition, there are theoretically promising properties of ampakines, such as regional specificity of induction, enhanced plasticity, and amplification of LTP. Altogether, this renders ampakines a potentially interesting class of drugs to explore in relation to implicit learning.

## 10. CONCLUSIONS

First a note of caution with respect to claims of dissociations between the neuropharmacology of implicit and explicit learning. Here, one must be careful not to over-interpret simple dissociations such as when explicit but not implicit learning is effected, for example by scopolamine [81, 82], since null effects might be related to low sensitivity in the experimental task chosen to probe a given learning system. Negative results might be interpretable, but this requires high sensitivity in the experimental task(s) in combination with receptor specific pharmacological agents. Preferably, well-characterized dose-response curves should be provided. Moreover, if additional measures of intervention effects, for example functional neuroimaging data, are acquired in addition to behavioral results, conclusions concerning regional specificity are easier to substantiate.

Concerning the neuropharmacology of implicit learning there are still large gaps in the published literature. However, we can already draw some tentative conclusions (for an overview of the reviewed results see Figure (**3**), Table **1** for human results and Fig. (**4**), Table **2**for animal results). It seems well-established that dopamine agonists/antagonists can modulate implicit and procedural learning in directions predicted by computational models of the basal ganglia. The effects of dopamine are most prominent during the early phases of acquisition and localized to the striatum, possibly the dorsal striatum [58-61]. Effects of dopamine on behavior have been observed also when there is no feedback provided [58-61]. There is some evidence for an involvement of serotonin on probabilistic learning in humans and avoidance learning in rats. Generally, the impact of serotonin on implicit learning is probably greater than that of acetylcholine. Acetylcholine has been demonstrated to have some effects on implicit learning, while the demonstrated effects of noradrenaline are scarce. The predictions from the reviewed computational models hold for both dopamine [39, 43-46] and serotonin [46, 49]. However, the evidence for noradrenaline and acetylcholine in implicit or procedural learning is too scant to draw any conclusions. One reason, why this might be the case, is the fact that unlike the dopamine and serotonin system, neither of these systems project directly to the striatum. The reviewed literature also suggests that the GABAergic system might be involved in implicit learning.

In general, further detailed investigations of neuropharmacological mechanisms by means of dose-response curves is an important next step that could support more firm conclusions, given that some of the present findings are mixed with respect to the direction of behavioral effects (i.e. whether performance enhancements or impairments are seen). Finally, ampakines are allosteric modulators of the AMPA receptors. Performance enhancements after ampakine administration has been demonstrated in a spatial maze task, associative memory and other memory tasks. This makes ampakines potentially interesting to investigate with respect to procedural and implicit learning.

## Figures and Tables

**Fig. (3):**
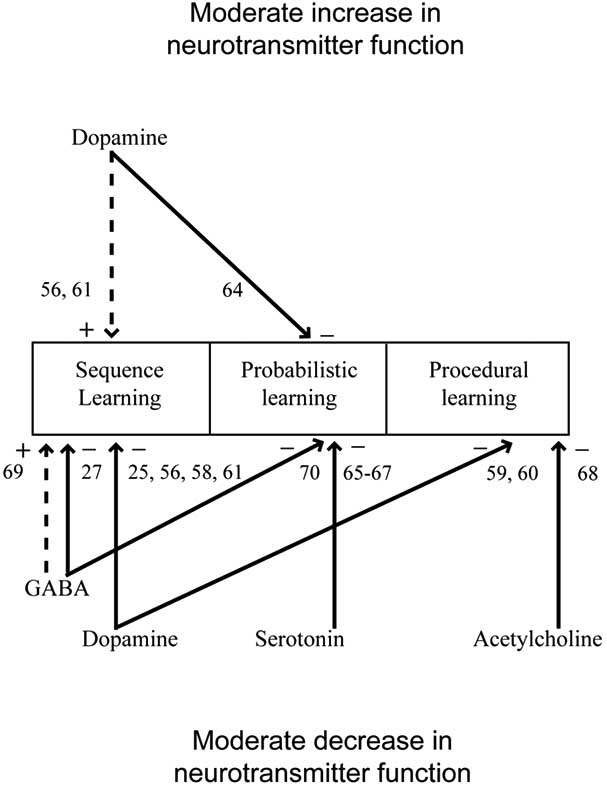
The figure presents an overview of the results on healthy volunteers and patients. From above, the arrows correspond to a manipulation with moderately increased neurotransmitter function. Arrows from below represent moderate decreases. At the arrow head, the direction of the performance change is indicated, with a plus for a performance increase and a minus for impaired performance. In addition, a dashed line indicates that there was a performance increase and a solid line that there was a performance decrease.

**Fig. (4):**
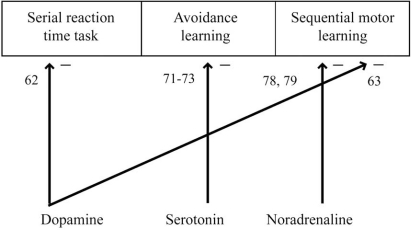
The reviewed rat data is summarized according to impaired performance in three varyingly precise categories of tasks: (1) an animal/rat version of the serial reaction time task; (2) avoidance learning; (3) sequential motor learning in general.

**Table 1.** Review of Human Data on the Neuropharmacology on Implicit Learning Tasks

Agent	Study	Modulatory Direction	Sequence Learning	Probabilistic Learning	Procedural Learning
Dopamine	[56, 61]	↑	↑	-	-
Dopamine	[64]	↑	-	↓	-
Dopamine	[25, 56, 58, 61]	↓	↓	-	-
Dopamine	[59, 60]	↓	-	-	↓
Serotonin	[65-67]	↓	-	↓	-
Acetylcholine	[68]	↓	-	-	↓
GABA	[69]	↓/↓	↓/↑	-	-
GABA	[70]	↓	-	↓	-

The table summarizes the reviewed articles by transmitter system, sorted on a moderately induced increase (↑) or decrease (↓) in neurotransmitter function. The most relevant findings are those that modify implicit learning of sequences, producing performance enhancements (↑) or interferences (↓). We have also reviewed studies of probabilistic learning (most relevant in connection to serotonin) and procedural learning of complex tasks (puzzles or tracking tasks).

**Table 2.** Review of Animal Data on the Neuropharmacology on Implicit Learning Tasks

Agent	Study	Modulatory Direction	Serial Reaction Time Task	Avoidence Learning	Sequential Motor Learning
Dopamine	[63]	↓/↓	-/↓	-	↓/-
Serotonin	[71-73]	↓	-	↓	-
Noradrenaline	[78, 79]	↓	-	-	↓

The table summarizes the reviewed articles on rats by transmitter system. In all of these articles, a moderate decrease (↓) in neurotransmitter function was induced. The most relevant findings for implicit learning are those that modify the serial reaction time task or other sequential motor learning tasks. All studies produced interferences with task performance (↓). We have also included studies of avoidance learning (most relevant in connection to serotonin). Those are standing in the avoidance learning column.

## ABBREVIATIONS

DA: = Dopamine
5-HT: = Serotonin
ACh: = Acetylcholine
GABA: = γ-aminobutyric acid
NMDA: = N-methyl-D-aspartic acid
AMPA: = α-amino-3-hydroxy-5-methyl-4- isoxazolepropionic acid
LTD: = Long term depotentiation
LTP: = Long term potentiation
AGL: = Artificial grammar learning
MTL: = Medial temporal lobe
VTA: = Ventral tegmental area
SRTT: = Serial reachtion time task
D1/D2: = Dopamine 1/dopamine 2 receptor
HVA: = Homovanillic Acid
SSRI: = Selective serotonin reuptake inhibitor
MDMA: = 3,4-methylenedioxymethamphetamine
BOLD: = Blood oxygen level dependent
FMRI: = Functional magnetic resonance imaging
BDNF: = Brain derived neurotrophic factor
EEG: = Electroencephalography
